# Epidermal growth factor signals regulate dihydropyrimidine dehydrogenase expression in EGFR-mutated non-small-cell lung cancer

**DOI:** 10.1186/s12885-016-2392-0

**Published:** 2016-06-06

**Authors:** Tetsuro Tominaga, Tomoshi Tsuchiya, Koji Mochinaga, Junichi Arai, Naoya Yamasaki, Keitaro Matsumoto, Takuro Miyazaki, Toshiya Nagasaki, Atsushi Nanashima, Kazuhiro Tsukamoto, Takeshi Nagayasu

**Affiliations:** Division of Surgical Oncology, Department of Surgery, Nagasaki University Graduate School of Biomedical Sciences, 1-7-1 Sakamoto, Nagasaki, Nagasaki 852-8501 Japan; Department of Surgery, Miyazaki University School of Medicine, 5200 Kihara, Miyazaki, Miyazaki 889-1692 Japan; Department of Pharmacotherapeutics, Nagasaki University Graduate School of Biomedical Science, 1-14 Bunkyo, Nagasaki, Nagasaki 852-8521 Japan

**Keywords:** Non-small-cell lung cancer, Sp1, Dihydropyrimidine dehydrogenase, Epidermal growth factor receptor mutation, 5-fluorouracil

## Abstract

**Background:**

It has been shown that epidermal growth factor receptor (EGFR) mutation status is associated with 5-fluorouracil (5-FU) sensitivity in non-small-cell lung cancer (NSCLC). However, the relationship between EGFR mutation status and dihydropyrimidine dehydrogenase (DPD), a 5-FU degrading enzyme, is unknown.

**Methods:**

We elucidated the crosstalk among the EGFR signal cascade, the DPD gene (*DPYD*), and DPD protein expression via the transcription factor Sp1 and the effect of EGFR mutation status on the crosstalk.

**Results:**

In the PC9 (exon19 E746-A750) study, EGF treatment induced up-regulation of both Sp1 and DPD; gefitinib, an EGFR-tyrosine kinase inhibitor (EGFR-TKI), and mithramycin A, a specific Sp-1 inhibitor, suppressed them. Among EGFR-mutated (PC9, HCC827; exon19 E746-A750 and H1975; exon21 L858R, T790M, gefitinib resistant) and -non-mutated (H1437, H1299) cell lines, EGF administration increased *DPYD* mRNA expression only in mutated cells (*p* < 0.05). Accordingly, gefitinib inhibited DPD protein expression only in PC9 and HCC827 cells, and mithramycin A inhibited it in EGFR-mutated cell lines, but not in wild-type. FU treatment decreased the level of cell viability more in gefitinib-treated EGFR-TKI sensitive cell lines. Further, combination treatment of FU and mithramycin A suppressed cell viability even in a gefitinib resistant cell line.

**Conclusions:**

The EGFR signal cascade regulates DPD expression via Sp1 in EGFR mutant cells. These results might be a step towards new therapies targeting Sp1 and DPD in NSCLC with different EGFR mutant status.

**Electronic supplementary material:**

The online version of this article (doi:10.1186/s12885-016-2392-0) contains supplementary material, which is available to authorized users.

## Background

The epidermal growth factor receptor (EGFR) is highly expressed in cancer cells, including non-small-cell lung cancer (NSCLC). Molecular-targeted therapy by EGFR tyrosine kinase inhibitor (EGFR-TKI) has altered the treatment regimen of NSCLC. *In vitro* and clinical studies have indicated predictive factors associated with the response and survival benefit of EGFR-TKI, including gefitinib, and higher response populations are observed in East Asians, non-smokers, females, and adenocarcinoma (ADC) patients [1–4]. A combination analysis of a Japanese phase II study found that NSCLC patients with EGFR mutations have higher response rates for EGFR-TKI [5]. Several randomized studies have also revealed that reversible EGFR-TKIs such as erlotinib and gefitinib are superior to standard chemotherapy with regard to progression-free survival, as well as progression-free survival in patients with NSCLC with EGFR sensitive mutations [6–10]. However, primary or acquired resistance limits the therapeutic success of these targeted agents [11, 12]. An irreversible inhibitor of afatinib has been developed to confer sustained disease control in ErbB-dependent cancers. A large LUX-Lung 3 phase III trial recently demonstrated that afatinib is clearly superior to the most effective platinum doublet in patients with EGFR mutation-positive lung cancer [13]. Thus, trials testing the potential efficacy of afatinib for reversible EGFR-TKI resistant NSCLCs are ongoing.

Anti-metabolite drugs, such as the fluoropyrimidine anti-cancer agent 5-fluorouracil (5-FU) and pemetrexed, have been used worldwide for chemotherapy with several types of solid tumor. Enzymes that participate in the pyrimidine metabolic pathway, including thymidylate synthase (TS), orotate phosphoribosyltransferase (OPRT), and dihydropyrimidine dehydrogenase (DPD), play an important role in FU sensitivity [14]. For pemetrexed, high TS has been shown to be correlated with resistance based on clinical and cell culture studies [15]. DPD is the initial enzyme in the pyrimidine catabolic pathway, and about 80 % of administered FU is rapidly catabolized in the liver [16–18]. Previously, the use of FU was thought to be inappropriate for lung cancer therapy, because the lung contains high levels of DPD, which is known to degrade FU. However, some DPD inhibitory fluoropyrimidines (DIF) have been developed which avoid the problem of degradation by DPD [19–22].

In 2007, Suehisa and colleagues showed that the IC_50_s of EGFR mutant cells to FU were higher than those of wild-type cells, indicating that EGFR wild-type cells are more sensitive to FU than mutant cells [23]. In our previous immunohistochemical staining for DPD in NSCLC specimens, high DPD expression was significantly correlated with EGFR mutation status and adenocarcinoma with a lepidic pattern [24]. Cell-based and histological studies led us to believe that EGFR mutation status might affect the sensitivity to FU due to high DPD expression in cells. Because EGFR-mutated NSCLC shows high DPD expression while EGFR wild-type shows low DPD expression, which correlate with EGFR-TKI and 5-FU sensitivity, we considered that there is a biological rationale for studying the interplay between EGFR mutation status and DPD expression for drug selection and personalized chemotherapy.

Accordingly, there are some molecular clues that explain the correlation between the EGFR cascade and DPD expression. *DPYD*, the DPD gene, has two transcriptional regulatory regions, and the binding of transcription factor specificity protein 1 (Sp1) to the *DPYD* promoter region is implicated in DPD constitutive expression [25, 26]. Lee and colleagues reported that Sp1 is present downstream of the EGFR-ERK1/2 signal cascade [27]. These studies indicate the possibility that Sp1 intermediates the EGFR-ERK1/2 signal cascade and DPD expression.

In the present study, we examined the signaling cascade from EGFR to DPD *in vitro.* To examine signal cascade regulation, we treated NSCLC cell lines with EGFR signal activator (EGF) or inhibitors (gefitinib, mithramycin A). In addition, we investigated whether EGFR mutation status affects the EGFR signal cascade, Sp1 regulation, and DPD expression.

## Methods

### Drugs

Gefitinib was purchased from Tocris Bioscience (Bristol, UK). Mithramycin A, a specific Sp1 transcription factor inhibitor, was obtained from SERVA Electrophoresis GmbH (Heidelberg, Germany). EGF and antibodies against ERK1/2, pERK1/2 (Phospho-Thr202/Tyr204), Sp1, and DPD were purchased from Cell Signaling (Beverly, MA, USA). β-actin (loading control) was from Abcam (Cambridge, UK).

### Lung cancer cell lines and culture conditions

Human lung adenocarcinoma cell line PC9 (exon19 E746-A750) was obtained from Immuno-Biological Laboratories (Gunma, Japan). NCI-H1975 (exon21 L858R, T790M), NCI-H1437 (wild-type), and NCI-H1299 (wild-type) were obtained from the American Type Culture Collection (Manassas, VA, USA). HCC827 was kindly provided by Dr. Isamu Okamoto (Kyushu University Hospital). These cell lines were incubated in a humid atmosphere containing 95 % air and 5 % CO_2_ in Roswell Park Memorial Institute (RPMI) media (Invitrogen Japan, Tokyo, Japan). The media contained 10 % fetal bovine serum, 1 % penicillin-streptomycin (Nacalai Tesque, Kyoto, Japan), and 2 mM L-glutamine (Invitrogen Japan).

### Quantitative polymerase chain reaction and mRNA expression

RNA in cultured cells was extracted using an RNeasy Mini Kit (QIAGEN, Tokyo, Japan), and cDNA was produced using PrimeScript RT Master Mix (Takara, Shiga, Japan). The sequences of PCR primers for mRNA in DPD, Sp1, and β-actin were as follows: DPD forward, 5’-GTTGTGGCTATGATTGATGA-3’, and reverse, 5’-ATTCACAGATAAGGGTACGC-3’; Sp1 forward, 5’-TTGAAAAAGGAGTTGGTGGC-3’, and reverse, 5’-TGCTGGTTCTGTAAGTTGGG-3’; β-actin forward, 5’-GCAAAGACCTGTACGCCAAC-3’, and reverse, 5’-CTAGAAGCATTTGCGGTGGA-3’. PCR reactions were performed with 20 ng of cDNA with LightCycler 480 SYBR Green I Master (Roche Molecular Biochemicals, Indianapolis, IN, USA). Quantitative RT-PCR was performed on a Roche LightCycler 480 system (Roche Molecular Biochemicals). Quantification data were analyzed with the LightCycler analysis software (Roche Molecular Biochemicals).

### Western blot analysis

Nuclear Extract Kit (Active Motif, Carlsbad, CA, USA) was used to collect whole cell lysates. Proteins were fractionated by sodium dodecyl sulfate polyacrylamide gel electrophoresis (SDS-PAGE) using 8 % gradient gels. The protein was blotted onto a polyvinylidene difluoride membrane. After blocking with Blocking One-P (Nacalai Tesque) for 20 min, the membrane was incubated overnight at 4 °C with primary antibodies against ERK1/2, pERK1/2, Sp1, DPD, and β-actin (loading control), followed by horseradish peroxidase-conjugated goat anti-rabbit IgG (Amersham, Buckinghamshire, UK). Immunolabeled protein was visualized using a Luminescent Image Analyzer LAS-1000plus (Fujifilm, Tokyo, Japan) after incubation with Chemi-Lumi One Super (Nacalai Tesque).

### EGF administration

To examine the relationship between DPD expression and the EGFR signaling cascade, we administered varying concentrations of EGF (0 to 10 ng/ml). After we grew NSCLC cells to 80 % confluence, culture medium was replaced with serum-free medium, and we then administered each concentration of EGF. After additional incubation at 37 °C for 20 min, cell lysates were extracted.

### Growth inhibition assay

The growth inhibitory activities of gefitinib and mithramycin A were evaluated by the cell proliferation reagent WST-1 (Roche Diagnostic GmbH, Mannheim, Germany). NSCLC cell lines were seeded in 96-well plates at a density of 10^5^/well with culture medium for 24 h. The cells were exposed to various concentrations of FU and gefitinib/mithramycin A for another 24 h at 37 °C in a 5 % CO_2_ atmosphere. After that, WST-1 was added to each well and incubated for 2 h at 37 °C before measuring absorbance at 490 nm with a Multiskan JX Spectrum instrument (Thermo Labsystems, Boston, MA, USA).

### Sp1 DNA binding assays

Cells were treated by gefitinib and mithramycin A, and nuclear proteins were extracted. Ten micrograms of nuclear extract were examined for Sp1 DNA binding activity by using a Trans AM Sp1 Kit (Active Motif), and control and treatment groups were compared. The absorbance was measured at 450 nm by a Multiskan JX Spectrum instrument (Thermo Labsystems).

### Apoptosis assay by DNA fragmentation

Apoptosis was evaluated by enzyme-linked immunosorbent assay (ELISA) using a Cell Death Detection ELISA^Plus^ Kit (Roche Applied Science, Mannheim, Germany). The amounts of mononucleosomes and oligonucleosomes generated from the apoptotic cells were quantified using monoclonal antibodies directed against histones and DNA by ELISA. Absorbance was measured at 405 nm by a Multiskan JX Spectrum instrument (Thermo Labsystems).

### Statistical analysis

Data from WST-1 assays and real-time RT-PCR are expressed as means ± S.D. The statistical significance of differences among the five cell lines was examined by Tukey-Kramer analysis. Student’s *t*-test and Dunnett’s multiple comparison were used to analyze associations between the control and experimental groups in each cell line. For all experiments, a two-sided *p*-value less than 0.05 was considered statistically significant. All experiments were performed in triplicate.

## Results

### EGFR signal cascade regulation on DPD protein expressions

**Fig. 1:**
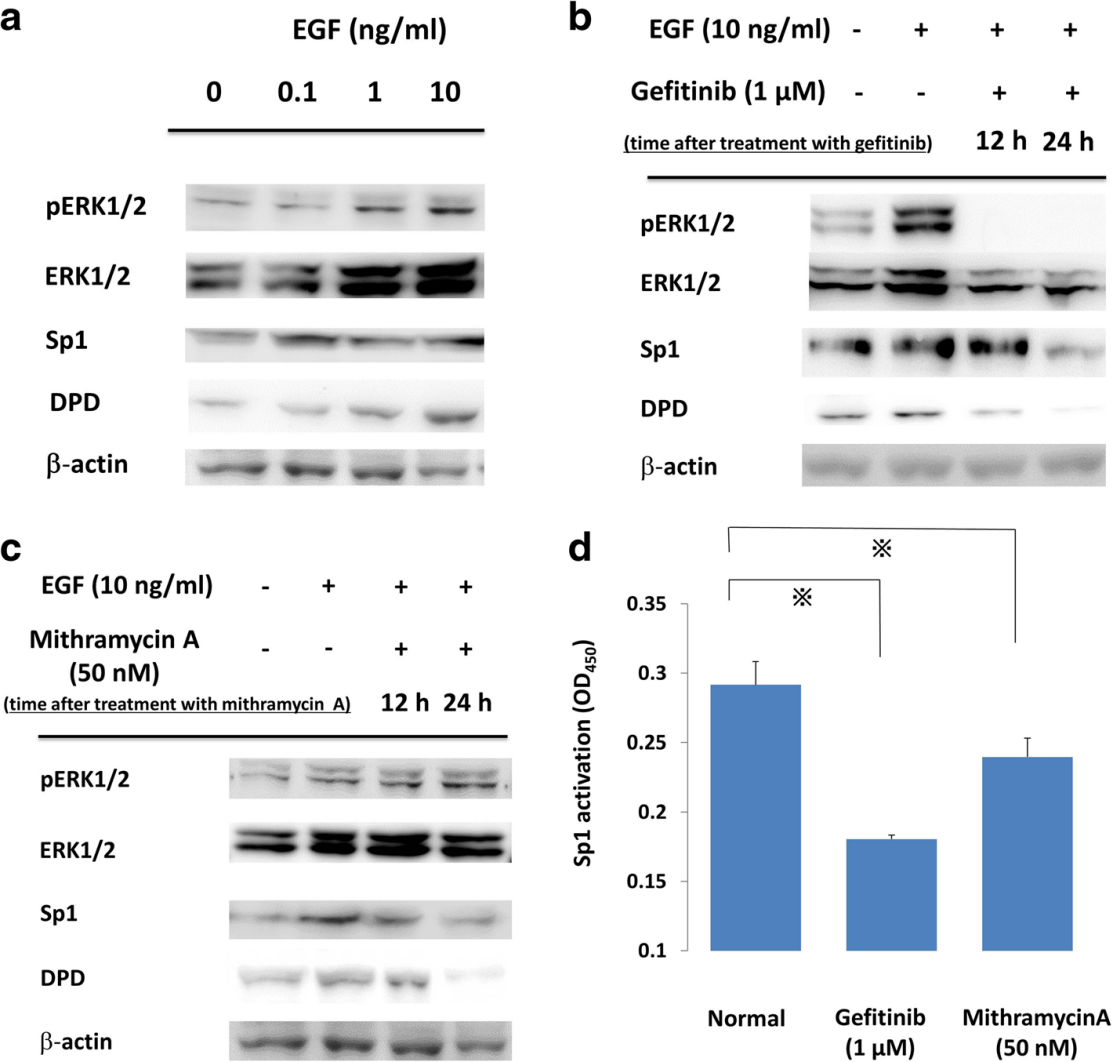
**a** PC9 cells were incubated with various concentrations of EGF, and whole cell lysates were extracted and were examined by immunoblot analysis. **b, c**. Effects of gefitinib and mithramycin A on ERK, Sp1, and DPD in immunoblot analysis. PC9 cells were pretreated with gefitinib (1 μM) or mithramycin A (50 nM) before administration of 10 ng/ml EGF. After treatment, whole cell lysates were extracted and examined by western blotting. Results are representative of two independent experiments. **d** PC9 cells were stimulated with inhibitors, and nuclear extracts were prepared to detect the Sp1/DNA interaction by a Trans AM Sp1 kit. The experiment was performed in triplicate. *, *p*-value < 0.05

To elucidate the relationship between the EGFR signal cascade and DPD expression, we first administered varying concentrations of EGF to PC9 cells (exon19 E746-A750). Western blotting showed EGF administration induced the phosphorylation of ERK1/2 and the expressions of Sp1 and DPD were increased dose-dependently (Fig. 1a). Second, we suppressed EGFR signal cascade by gefitinib, an EGFR-TKI. Western blotting showed that phosphorylations of ERK1/2 were induced by EGF stimulation and were suppressed rapidly by gefitinib, and EGF-induced expressions of both Sp1 and DPD were also suppressed in a time-dependent manner (Fig. 1b). To find out whether a reduction of Sp1 transcriptional activity affected DPD expression, PC9 cells were treated with mithramycin A, which is an inhibitor of Sp1. Western blot analysis showed that mithramycin A inhibited both Sp1 and DPD protein levels; however, the upstream signaling pathways, including ERK1/2 and pERK1/2, were not suppressed (Fig. 1c).

### Effect of EGFR-TKI and mithramycin a on Sp1 transcription activity

To test the effect on Sp1 transcriptional activity, PC9 cells were treated with gefitinib and mithramycin A. Extracted nuclear protein was examined for its Sp1 DNA binding activity by using a Trans AM Sp1 Kit. Sp1 transcription activity was suppressed compared with the baseline activity level using the two drugs (*p* < 0.05) (Fig. 1d).

### Comparison of the EGF effect in EGFR-mutated and -non-mutated NSCLC cell lines

**Fig. 2:**
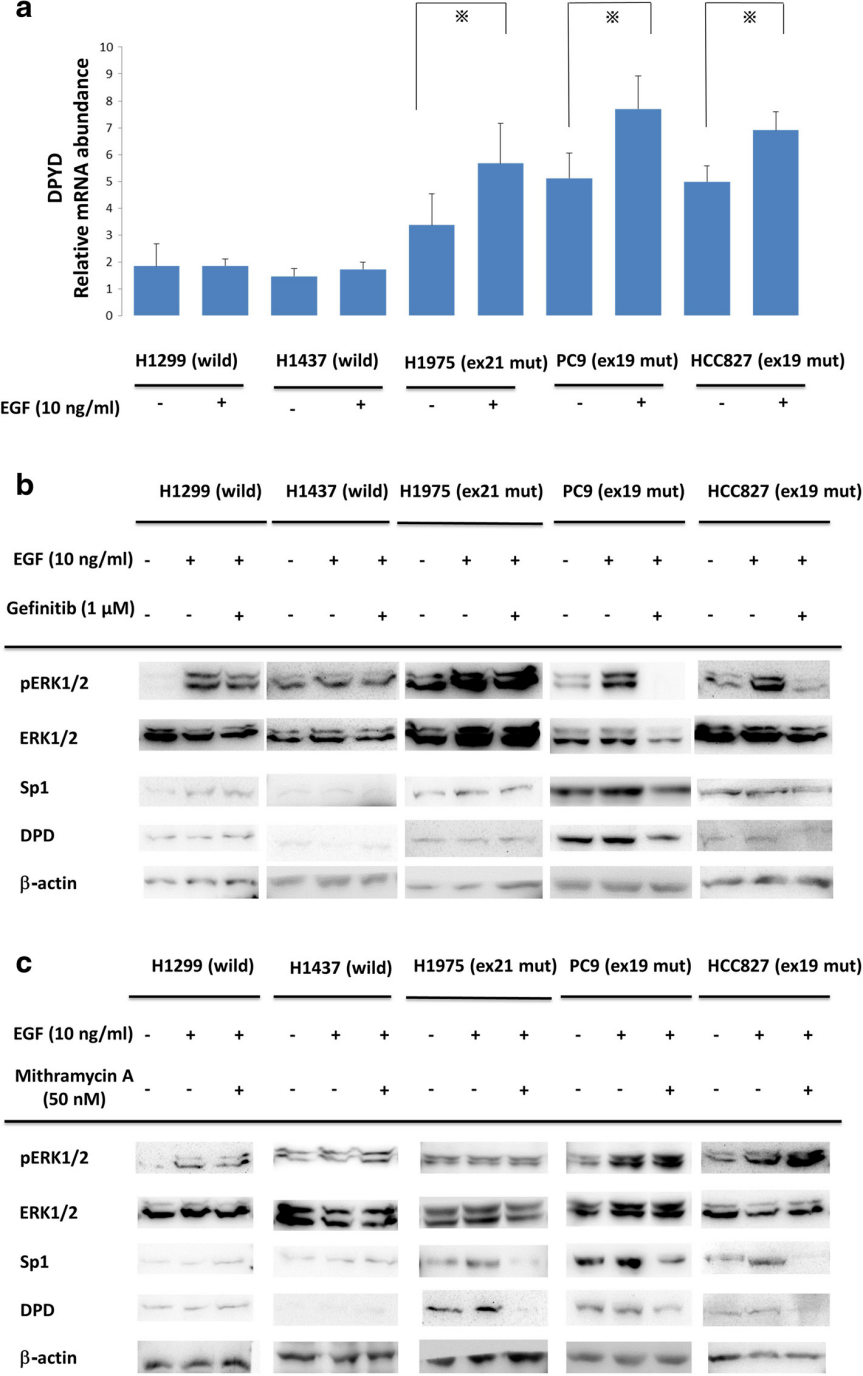
**a** Comparisons of *DPD* mRNA expression with EGF administration in NSCLC cell lines. Serum-free cells were incubated in the presence or absence of 10 ng/ml EGF. After that, *DPD* mRNA levels were quantified by RT-PCR. The experiment was performed in triplicate. *, *p* < 0.05 **b, c** NSCLC cell lines were treated with gefitinib (1 μM) or mithramycin A (50 nM) for 24 h followed by stimulation with 10 ng/ml EGF for 20 min. Extracted lysates were examined by western blotting. Results are representative of two independent experiments

To compare the effect of EGF in differential mutant types of NSCLC cell lines, EGF of 10 ng/ml was administrated to each cell line for 20 min (Fig. 2a). In EGFR mutant NSCLC cell lines (PC9, HCC827, and H1975), *DPYD* mRNA levels were significantly increased after EGF administration (*p* < 0.05). On the other hand, *DPDY* mRNA levels of EGFR in the wild-type cell lines (H1437, H1299) were not influenced by EGF treatment.

### Comparison of the Gefitinib or mithramycin a effects in EGFR-mutated and -non-mutated NSCLC cell lines

We next investigated the effects of signal cascade inhibitors gefitinib and mithramycin A in EGFR-mutated and -non-mutated NSCLC cell lines. NSCLC cell lines were treated with gefitinib (1 μM) or mithramycin A (50 nM) for 24 h followed by stimulation with 10 ng/ml EGF for 20 min. As shown in Fig. 2b, gefitinib treatment suppressed EGF-induced ERK1/2 phosphorylation and both Sp1 and DPD expression only in gefitinib-sensitive NSCLC cell lines PC9 and HCC827. H1975 has an acquired resistance mutation, T790M, against gefitinib treatment.

Figure 2c shows that ERK1/2 and pERK1/2 upstream signals of the EGFR cascade were not affected by mithramycin A. Interestingly, Sp1 and DPD were inhibited only in EGFR mutant cells.

### Comparison of apoptosis after treatment with Gefitinib/mithramycin a in EGFR-mutated and -non-mutated NSCLC cell lines

**Fig. 3:**
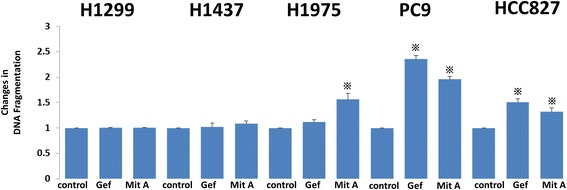
Apoptosis was evaluated by ELISA using a Cell Death Detection ELISA^Plus^ Kit (Roche Applied Science). To evaluate the effect of gefitinib and mithramycin A on cell apoptosis, we treated cells with gefitinib (1 μM) and mithramycin A (50 nM) for 24 h. Extracted cell lysates were evaluated by ELISA. In wild-type cells (H1299, H1437) there was little effect of the inhibitors on apoptosis. By contrast, apoptosis increased in EGFR-mutated cell lines treated with gefitinib, except for H1975 with the T790M mutation. Mithramycin A increased apoptosis in all EGFR-mutated cell lines. Gef, gefitinib; Mit A, mithramycin A. The experiment was performed in triplicate. *, *p* < 0.05

To evaluate the effect of gefitinib and mithramycin A on cellular apoptosis, we treated cells with gefitinib (1 μM) and mithramycin A (50 nM) for 24 h. Extracted cell lysates were evaluated by ELISA using a Cell Death Detection ELISA^Plus^ Kit. In wild-type cells (H1299, H1437) the inhibitors had little effect on apoptosis. In contrast, the apoptotic reaction increased in EGFR-mutated cell lines treated with gefitinib, except cell line H1975 with the T790M mutation. Mithramycin A increased apoptosis in all EGFR-mutated cell lines (Fig. 3).

### Comparison of the growth inhibition after combination treatment with FU and Gefitinib/mithramycin a in EGFR-mutated and -non-mutated NSCLC cell lines

**Fig. 4:**
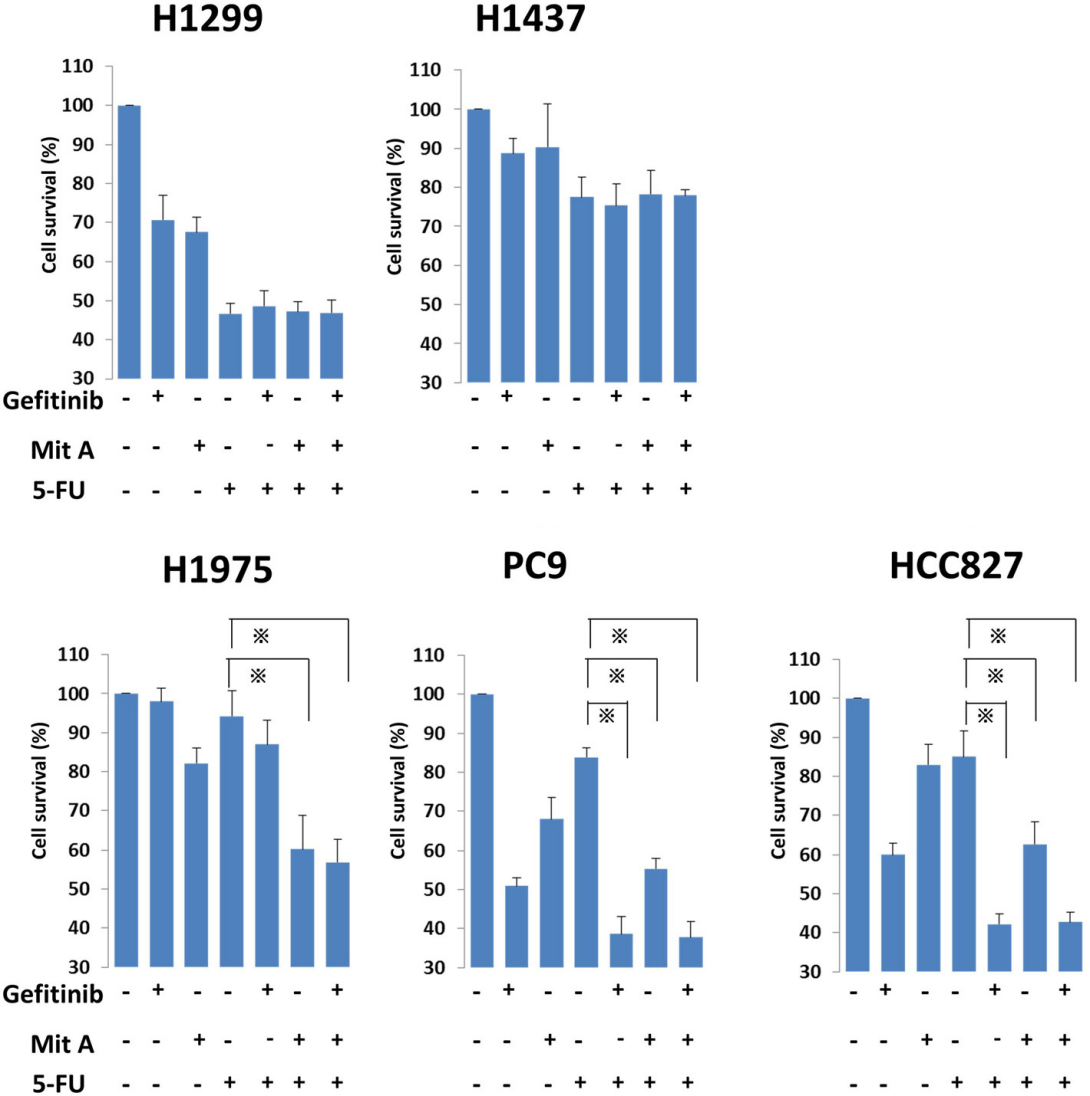
In combination assays, NSCLC cell lines were seeded in 96-well plates at a density of 10^5^/well with culture medium for 24 h. The cell lines were treated with FU (10 μM) alone or FU plus gefitinib (1 μM)/mithramycin A (50 nM) for another 24 h at 37 °C in a 5 % CO_2_ atmosphere. After that, WST-1 was added to each well and incubated for 2 h at 37 °C before measuring absorbance at 490 nm with a Multiskan JX Spectrum instrument. The experiments were performed in triplicate. * *p* < 0.05. Mit A, mithramycin A

To elucidate the effect of gefitinib or mithramycin A with FU in each cell line, we treated cells with combinations of FU (10 μM) and gefitinib (1 μM) or mithramycin A (50 nM). In PC9 and HCC827 cells, the levels of cell viability were significantly lower in the combination treatments including gefitinib and mithramycin A compared with FU treatment alone (Fig. 4). In H1975 cells, which have the T790M mutation, the IC_50_ values were lower only for the combination of mithramycin A and FU. The other two wild-type cell lines showed no differences when combining drugs. Cell viabilities using 5-FU, gefitinib, and mithramycin A were not different from the combination of two drugs (5-FU and gefitinib, or 5-FU and mithramycin A).

## Discussion

The present study clearly showed crosstalk among the EGFR cascade, transcription factor Sp1, and DPD expression in NSCLC cell lines. Using PC9 cells, EGF administration significantly increased DPD protein levels in a dose-dependent manner. Accordingly, gefitinib, an EGFR-TKI, inhibited the phosphorylation of ERK1/2 and decreased ERK1/2, Sp1, and DPD protein levels. The regulation of DPD by the above-described EGFR signal induction and inhibition indicated that the EGFR signal cascade directly regulates DPD expression. On the other hand, mithramycin A, a G-C specific DNA binding drug that can bind the consensus sequence of the Sp1 binding element [28], decreased Sp1 and DPD protein levels without inhibiting the phosphorylation and protein levels of ERK1/2. Therefore, we assessed that Sp1 is a principal candidate for the EGF-induced transcriptional regulator of DPD.

The activation of EGFR to the DPD cascade is different between EGFR-mutated and -non-mutated cell lines; EGFR mutant cells cause both ligand-independent and ligand-dependent activation of Sp1 to DPD signals. Consistent with a report by Sordella and colleagues showing that the receptor and downstream signaling are constitutively activated only in EGFR mutant cells [29], both Sp1 and DPD proteins were increased in EGFR-mutated cell lines compared to the wild-type under normal conditions (Fig. 2a). In addition, EGF administration increased *DPYD* mRNA expression only in EGFR-mutated cells. These results support the theory that EGF treatment induces higher and more consistent activation in mutant EGFRs than in wild-type EGFRs in NSCLC cell lines [1, 30]. Consistently, western blot analysis showed that gefitinib decreased ERK1/2 signaling with the suppression of the protein levels of Sp1 and DPD only in EGFR-mutated cells (Fig. 2b, c). In addition, the usage of mithramycin A showed that DPD suppression only occurred in EGFR-mutated cells, but not in wild-type cells. The two drugs also increased apoptosis in EGFR mutant cell lines (Fig. 3).

In this study, we examined H1975, another EGFR-mutated cell line that contains the T790M mutation and is resistant to gefitinib treatment (Additional file 2: Table S1). As expected, in western blot analysis, gefitinib did not decrease the DPD expression. Instead, mithramycin A clearly inhibited Sp1 and DPD expressions. Accordingly, in drug combination assays, gefitinib and FU showed additive anticancer effects in EGFR-mutated PC9 cells, but not in EGFR-resistant H1975 cells. On the other hand, mithramycin A and FU showed additive anticancer effects in all EGFR-mutated cells regardless of the T790M mutation.

The present results elucidated a relationship between clinical outcomes and the biology of EGFR-mutated NSCLC. First, in our previous histopathological study, we showed that adenocarcinoma *in situ*, previously classified as bronchioloalveolar carcinoma, has significantly higher EGFR mutation frequency and *DPYD* mRNA levels than other histological types [24]. The presently identified signal regulation supports our previous proposal that invasive adenocarcinoma and adenocarcinoma *in situ* might have different biological properties due to different EGFR signal cascade regulation and DPD activity [24]. Second, the relationship between EGFR mutation status and high DPD expression might serve as a predictor of drug sensitivity. Because DPD is a 5-FU degradation enzyme, EGFR-mutated tumors might resist DIF because of high DPD. Conversely, EGFR wild-type tumors might be sensitive to DIF because of low DPD. Consistent with this, it has been reported that EGFR wild-type tumors have higher sensitivity to uracil-tegafur than EGFR mutant tumors [23]. Third, the present results might affect the treatment strategy for EGFR-TKI-resistant tumors. Gefitinib and 5-FU additively reduced PC9 and HCC827 cell survival, suggesting that gefitinib treatment might increase the effect of 5-FU on EGFR-TKI-sensitive tumors, possibly from DPD suppression via EGFR signal cascade inhibition. In addition, mithramycin A could enhance the 5-FU effect on EGFR-TKI-resistant tumors via direct Sp1 suppression. Mithramycin A and 5-FU-related drugs might be another option for EGFR-TKI-resistant NSCLC treatment.

The limitation of the present study is that the cis-regulatory element of DPD contains several transcription factor-binding sites aside from Sp1, including AP, Egr, and NF-kB [26]. Fig. 4 shows that cell viability after treatment of 5-FU plus gefitinib was relatively high compared with treatment of 5-FU plus mithramycin A in PC9 and HCC827 cells. This implies the existence of some unknown transcriptional regulation present in this cascade, especially in EGFR mutant-type tumors (Additional file 1: Figure S1). Further analysis of other transcriptional regulation mechanisms might be necessary to elucidate the mechanisms of EGFR signal regulation for DPD expression. In the wild-type cell lines, 5-FU monotherapy did not affect cell survival in H1437 cells, even with low expression of DPD. In fact, the IC_50_ with 5-FU was higher in H1437 than in H1299, even though they have the same EGFR wild-type status (Additional file 2: Table S1). This is unsurprising because H1437 has the MEK1 exon2 mutation (Q56P substitution) [31], and MEK-1 signal suppression increases 5-FU sensitivity [32]. These reports and the present results indicate that continuous MEK-1 expression in H1437 results in 5-FU resistance, possibly because of a DPD-independent mechanism in this wild-type cell line.

## Conclusion

In conclusion, we have shown crosstalk among the EGFR cascade and Sp1 and DPD expressions in EGFR mutant cell lines. EGF-induced Sp1 up-regulation resulted in both *DPYD* mRNA and DPD protein expressions. Combination chemotherapy of 5-FU and EGFR-TKI additively suppressed cancer cell survival in EGFR-mutant cell lines. In particular, 5-FU and mithramycin A induced cytotoxicity in EGFR-resistant cell lines. Further studies are necessary to elucidate the detailed mechanism of EGFR cascade regulation of DPD expression, which may lead to new therapeutic strategies against NSCLC.

## Abbreviations

5-FU, 5-fluorouracil; DIF, DPD inhibitory fluoropyrimidines; DPD, dihydropyrimidine dehydrogenase; EGFR, epidermal growth factor receptor; EGFR-TKI, EGFR tyrosine kinase inhibitor; Mit A, mithramycin A; NSCLC, non-small-cell lung cancer; OPRT, ortate phosphoribosyltransferase; Sp1, specificity protein 1; TF, transcription factor; TS, thymidylate synthase

## Additional files

Additional file 1: Figure S1. Schematic diagrams of the signal cascade of EGF-induced DPD expression of EGFR-mutated type cells. TF, transcription factor; Mit A, mithramycin A. (JPG 130 kb) Additional file 2: Table S1. Effects of 5-FU, gefitinib, and mithramycin A on cell proliferation in NSCLC cell lines. Cells were incubated with various concentrations of 5-FU for 72 h, and cell viability was examined by WST-1 assay. The IC_50_ values of these drugs were calculated for each cell line. Cell viability of the control group (DMSO) was set at 100 %. Values are means from three samples from one representative experiment. (XLSX 9 kb)
