# Supplementary figures and images for: Epidermal growth factor signals regulate dihydropyrimidine dehydrogenase expression in EGFR-mutated non-small-cell lung cancer
Source: BMC Cancer. 2016 Jun 6;16:354. doi: 10.1186/s12885-016-2392-0 (PMC4896005; doi:10.1186/s12885-016-2392-0)

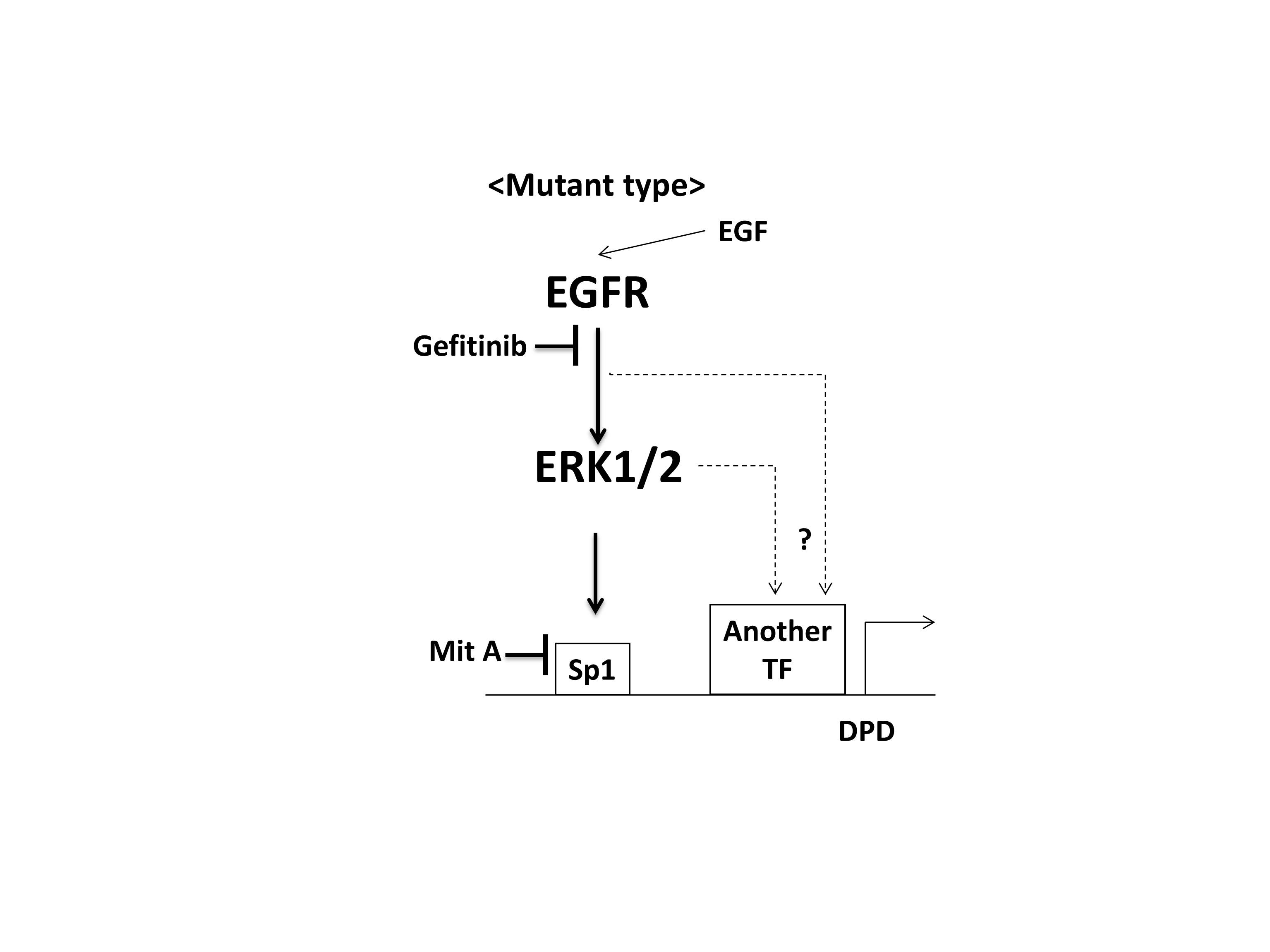

Supplement: Additional file 1: Figure S1. — Schematic diagrams of the signal cascade of EGF-induced DPD expression of EGFR-mutated type cells. TF, transcription factor; Mit A, mithramycin A. (JPG 130 kb) [file 12885_2016_2392_MOESM1_ESM.jpg]
